# Considering Culturally Sensitive Dementia Care as a Prerequisite for Person-Centered Care: The Turkish Community in Germany as an Example

**DOI:** 10.1007/s10728-025-00529-y

**Published:** 2025-06-18

**Authors:** Zümrüt Alpinar-Segawa, Sabine Salloch

**Affiliations:** Institute for Ethics, History and Philosophy of Medicine, Hannover Medical School, Hannover, Germany

**Keywords:** Culturally sensitive care, Dementia, Germany, People with migration background, Person-centered care, Turkish community

## Abstract

Culturally diverse societies often struggle with providing appropriate dementia care. Cultural sensitivity is considered an important prerequisite for meeting the needs of persons with dementia. This article discusses culture specific aspects of dementia care by referring to the Turkish community in Germany as an example. Factors are discussed that specifically infringe on the quality of dementia care for migrants. The article defends the claim that good dementia care for migrants can be provided through a person-centered approach which is again based on culturally sensitive approach. We show how culture shapes health phenomena but also argue that a focus on culture may stereotype individuals as belonging to a particular culture, grouping people together irrespective of their heterogeneity. Person-centered care is ideal for recognizing diverse needs and values. It is often seen as being at odds with culturally sensitive care, but this paper suggests a way of reconciling them. We argue that culture does indeed provide a framework to create the necessary foundation for person-centered care. Finally, some criticisms and plausible replies are discussed and practical implications arising from the analysis are presented.

## Dementia Care: The Example of Turkish Migrants in Germany

Individual and family immigration brings cultural diversity to healthcare systems. As reported in World Migration Report 2022 [1], Germany has the highest immigration in Europe, with almost 16 million migrants in 2020. This creates “the largest foreign-born population of any country in Europe” ( [1], p. 88). 22.3 million people living in Germany, which is 27.3 per cent of Germany’s population, have a migrant background, meaning that they do not have German citizenship from birth, or have at least one parent who does not have German citizenship from birth [2]. Roughly 2.7 million individuals have a Turkish migration background, constituting the largest migrant group in Germany^1^ [2].

There are currently around 1.6 million people living with dementia^2^ in Germany, of which approximately 100.000 people have a migration background [5]. The first-generation Turkish migrants (i.e., who were born in Turkey and have migrated themselves) typically came to Germany due to labor migration in the 1960s. This population has now reached an age at which the susceptibility to dementia is higher [6]. In Germany, however, the potential for family care is likely to diminish in the future as the number of working women with a Turkish migrant background continues to rise and the number of members of the younger generation declines [7]. This situation is likely to create more dependency on seeking help outside the home and in health facilities. This group, therefore, represents a large potential group for dementia-related healthcare services [8].

In culturally diverse societies, access to appropriate health services and the responsiveness of those services to the needs of the individual are often challenging [6]. Many factors influence inadequate access to care, including socio-economic background, immigration status, language skills, stigma and marginalization. If the health services are insensitive to these issues, perceptions of discriminatory behaviors will be reinforced, and, if these perceptions become widespread, may lead to a decrease in trust in health services and help-seeking behavior, and postponement of care [6, 9–11]. Ideally, individual-centered care guided by the principle of respect for persons would be sufficient to provide appropriate care by recognizing diverse individual needs and values. In practice, however, this is not the case, specifically for people with migration background, as there are gaps for minority groups in terms of access to dementia care, knowledge about the disease, and how to deal with practical issues related to dementia care and life planning (see [6, 12–15]). There is currently a growing emphasis on the need for timely dementia diagnosis and proper care for people from different cultural backgrounds [16]. However, migration is underrepresented in national dementia plans across Europe, and there are a lack of services and guidelines to address the needs of people with migration backgrounds [17].

This paper will defend the claim that good care for migrants with dementia can be provided through a personcentered approach, the basis of which is the culturally sensitive approach. Given the heterogeneity of people, and the diversity of values and needs, it is important to identify individual needs while not ignoring the cultural background of the person. The paper begins by exploring the culture-specific aspects of dementia care. This will be followed by an explanation of culturally sensitive care and person-centered care^3^. After stating that they are complementary, the influence of culture on the shaping of health phenomena will be discussed. It is then argued that a person-centered approach to care combined with the culturally sensitive approach is necessary to provide good care to culturally diverse communities. Finally, some potential criticisms will be anticipated and plausible replies will be given.

### Culture Specific Aspects of Dementia Care

Dementia care choices are influenced by having a cultural background that can relate to individualistic and collectivistic characteristics [18], for example. Having an individualistic background reinforces acts based on personal perceptions and values as well as a more independent understanding of the self, whereas having a collectivistic background makes group or community important and values an interdependent self. Therefore, collectivistic-oriented people might tend to choose family care rather than institutionalizing the family member with dementia, which is reinforced by the culture-value-laden conceptions of responsibility for elderly members or filial duty; that is, an obligation that should not be failed [18]. This may also be related to the influence of culturally shaped ideas about family responsibilities and the good life on caring for those with dementia. Some studies have shown the importance of family care by finding that most elderly people with a Turkish migration background are cared for by their families, which is seen as a filial duty [6, 15, 19–21]. Some other studies conducted in Europe have also shown that immigrant groups (particularly non-Western immigrants) are less likely to be placed in a nursing home than the native population [22]. Although we cannot claim that group members having the same cultural background make identical care choices, it was found that due to cultural and language barriers, as well as a lack of migration-sensitive care, a low level of use of professional healthcare services among migrants from Turkey was found [19].

Having a migration background is associated with certain care needs [7]. Even though dementia is a challenging syndrome, both for the person with dementia and for their family members, regardless of migration background, further challenges are present for people with migration background: stigmatization and tabooing of the syndrome, which was observed among migrants from Turkey, loss of the learned language ability and the migration experience itself [15, 23].

Stigmatization and tabooing of the syndrome shape attitudes towards health (e.g., dementia comes naturally to everyone as part of aging), sickness (e.g., if no one mentions it, it will be forgotten and accepted as normal, or the types of dementia are not illness at all) and care (e.g., management of an illness, hiding the illness, keeping it as a secret in the family, only family should take care, etc.). It is a barrier to help-seeking behavior. Some empirical studies conducted with the family caregivers of people with dementia with Turkish migration backgrounds showed the need for information and support, as well as lack of knowledge concerning dementia and its progress [6, 20]. Although more empirical research is needed to show whether there is such a strong cause-and-effect relationship between lack of knowledge and tabooing, it is particularly difficult to reach this population [6, 24].

Missing language is a barrier to communication and it becomes apparent with advanced dementia, as the person with dementia’s ability to talk decreases. There is a further difficulty if the person with dementia has learned the language she is expected to communicate with, for instance for a person with a migration background. As the disease advances, the ability to use the learned language decreases, which creates shame for the person as well, and the person sticks to her mother tongue [23]. This brings about certain requirements for care of people with a migration background. For instance, a study showed that sharing a similar socio-cultural background, which allows familiarity and emotional closeness, and speaking the same language (i.e., a common mother tongue) were among the prerequisites of good care for elderly migrants from Turkey [19]. Furthermore, having a migration experience (i.e., living in a foreign country as a migrant, changes in living and working conditions, facing stressful and disadvantaged situations, etc.) can affect one’s mental health and initiate cognitive decline, which make migrants need care earlier than the native population (see [6, 25]– [26]).

The inadequacy of services is argued to be due to linguistic, cultural, religious and ethnic differences between the dominant culture and people with migration backgrounds [11, 25]. However, the effectiveness of healthcare is not only due to lack of information, language and communication difficulties on the part of individuals, but also a lack of sensitivity on the part of healthcare professionals to cultural differences, for example, a different understanding of illness and a different way of dealing with it (see [27]). It is important to recognize that some people with migration backgrounds have grown up in different cultures; meaning they have different cultural backgrounds. Considering the special case of dementia, migration and cultural differences, cultural sensitivity is becoming increasingly important in dementia care. However, it is unclear what culturally sensitive care means and what it entails.

### Culturally Sensitive Care and Person-Centered Care: Different or Complementary?

Culture is often described as “a closed and coherent system of individual societies” ( [28], p. 7, authors’ translation), as standards of patterns of behavior and a system of beliefs that one acquires as a member of a particular group [9, 29]– [30]. Culture provides a meaning-imparting context for the range of options people have for implementing their values and preferences [31]. This allows one to form and revise a personal conception of a good (or fulfilled) life [32]. Culture “provides its members with meaningful ways of life across the full range of human activities, including social, educational, religious, recreational, and economic life, encompassing both public and private spheres” ( [31], p. 76).

Cultural membership shapes one’s self-identity and provides a sense of belonging [31]. Culture provides the conceptual framework that determines which values, life plans or aims inform how a person’s conception of good life and self-identity is generated. Thus, insofar as conceptions of a good life inform an individual’s health(care) choices, culture is remarkably important for analyzing dementia-related care choices.

Culture shapes one’s expectations regarding good care, because it plays a significant role in shaping persons’ attitudes towards risky and healthy behaviors and in influencing decision-making with respect to care and treatment. Thus, culture has an impact on persons’ perceptions of illness, health, responsibility and obligations towards one’s family, etc [33–38]. For instance, there are studies documenting cultural differences in intergenerational relationships, including the willingness to care for parents (see [39]).

If culture is important and forms one’s expectations of good care, to provide appropriate care healthcare personnel should take into consideration cultural background and culture-specific aspects. This implies that healthcare services should be culturally sensitive. In the literature, culturally sensitive care is defined as being aware of and respecting cultural values and beliefs, being open to wishes and decisions, showing tolerance, empathy and interest in the background of the other and putting effort into understanding each other [40–42]. It is argued that this can be achieved by training healthcare personnel on cross-cultural competence^4^ [41]. This approach aims to increase patient satisfaction by improving the quality and outcomes of healthcare services [6, 34, 36].

In the literature, another promoted approach is that of person-centered dementia care, which emphasizes that care should pay attention to and value an individual’s perspectives, personality, subjectively defined experiences and needs, and their network of relationships in providing care [43]– [44]. An understanding of life and identity of the person with dementia would increase the quality of care. According to Kitwood, maintaining personhood and well-being through the interaction between the care provider and the person with dementia is crucial to providing good dementia care [45], which is the basis of person-centered care. For people with chronic illness and disability and in need of long-term care, person-centered care gains importance to improve the quality of care, as it focuses on recognition of each individual with distinct preferences and needs [46]. However, “little research has been conducted to explore the meaning of PCC [patient-centered care] for individuals at different stages of dementing illness, or for individuals varying by gender, age or ethnicity.” ( [43], p. 17). Person-centered care requires more effort and time investment from healthcare personnel, in terms of continuity, assignment of one caregiver to one family, and exploring the needs of the persons with dementia and their families, providing care to the whole family, as it means actively seeking and supporting one’s concept of self, identity and personhood [47]– [48].

By focusing on the individual person and his or her biography, belonging to a (cultural) group tends to become less significant. The individual would then be understood as a single person with his or her values, preferences, etc. In this sense, person-centered care would promote a narrow approach to care. On closer examination, however, it becomes clear that a person’s individual characteristics are, of course, very much shaped by the environment in which he or she grows up and the circumstances under which he or she lives. From this perspective, person-centered care cannot dispense with the need to take cultural aspects into account. Some tension remains between the individual as a person and as a member of a (culturally-shaped) group. It should be mentioned that there are some approaches, which tend to shift the focus from the individual to a broader context including the values of the individual’s family and cultural background of the individual, which would require a detailed biographical work (see [47]). It is also important to emphasize individual needs rather than limiting them to one’s own culture, bearing in mind that families and individuals are heterogeneous and not homogeneous within a community [23]. “Dementia care services need to be transparent and ‘culture-, religion- and migration-sensitive’ *whilst still maintaining an individualized care strategy*” ( [41], p. 1760, emphasis added). In order to provide good care, it is therefore important to identify the needs of the individual without ignoring the person’s cultural background.

### Culture Forms Approaches to Good Care

Culture is important in shaping one’s health phenomena, influences the person’s understanding of illness, good life and shapes (health) attitudes. Culture gives us a framework to provide the base required for person-centered care. Culture is about the way we grew up, our habits, what we are used to, etc. Family, and the environment we grew up in both play a role in shaping our expectations regarding good life, good care, understanding of illnesses, expression of pain, etc., even though these might show differences on a personal level.

We can think of culture in two layers that form an individual person’s values and needs: (1) The general culture that a person was born into and which surrounds the person (i.e., the basic structure shaping the person’s attitudes and perspectives), and (2) one’s biographical background (i.e., their personal level of development filtered through family, social environment, life philosophy). While the first layer covers a broad and superfluous range of qualities, such as the commonalities shared with others (e.g., an understanding of what is expected in relation to public holidays and religious festivals, food culture, media, values, manners and customs, history, etc.), the second refers to a more personal level, such as family (e.g., family values, how one is raised, etc.), social environment (e.g., socio-economic condition, friends, etc.), the education one received, life philosophy (e.g., personal experiences, ideals, being faithful or not, etc.). If we think of the first layer as a ground layer or as a roof, i.e., the basics, the second layer would determine more specifically the person’s needs on a personal level. A combination of these two layers could help us to understand what the expectations are in terms of care.

What would that mean to ensure that good care is provided? Professional caregivers should be familiar with the cultural background of the person with dementia. It is not enough to have the basic communication skills, openness and empathy that are the required qualities of a good caregiver, but familiarity is also necessary to ensure good dementia care. Dementia is a special case because persons with dementia often primarily remember the early parts of their life. Let’s take a first-generation Turkish migrant, Ahmet, who came to Germany as a guest worker in his early 20s. Ahmet developed Alzheimer’s disease and his condition progressed. He has perfect recall of his childhood memories, songs and tastes of food, but cannot recall more recent memories. Although he could speak German, he can no longer speak German. Ahmet grew up in a small village in south-west of Turkey (environmental layer of culture). As a child, he used to bathe in a basin as a child, where he poured water with a jug. The smell of tarhana soup^5^ warms him up and makes him feel comfortable and safe (biographical layer of culture). A responsive care requires biographical work, but also a familiarity with the culture. If the caregivers are not familiar with this concept of bathing, which was used to be quite common in small villages, or this soup, which is peculiar to Turkish cuisine, they will be confused and unable to provide the care that Ahmet needs.

The Alzheimer’s Association has identified holistic, person-centered dementia care as the foundation of quality care for people with dementia [44]. However, the practice recommendations for dementia care do not place as much emphasis on the influence of culture on care and are limited to culturally appropriate food choices or culturally sensitive training programs for professionals. However, the conception of culture in layers presented here keeps the focus on the individual without separating him or her from the cultural background that shapes himself or herself. Both these layers need to be considered to ensure that good care is provided.

Culture is an important aspect in providing appropriate care and cannot be ignored. However, we need to be aware of the pitfalls that can be created by referring to culture in order to achieve good care. Culture might imply grouping people together under one label or box sharing the same background, which might reinforce stereotyping or wrong assumptions regarding needs and expectations, irrespective of the variations within a group [49–52]. Irrespective of people’s migration background, individuals differ in terms of levels of education, sexual orientation, gender, religious affiliation, biographical background, etc. What is more, people sharing the same cultural background can have diverse values due to acculturation and higher levels of education of younger generations [53] as well as due to globalization and cultural mobility [54]. Besides, culture has a dynamic character; meaning it has a procedural character and does not stand still:

It is also important to be aware of the processual nature of cultures, because every culture is capable of change; after all, a culture is an adaptation of people to the respective circumstances and these are constantly changing ( [40], p. 143, authors’ translation).

Culture’s dynamic character hangs on factors, such as increasing levels of (higher) education, mobilization of people, widespread use of the internet and acquiring of knowledge, etc. These change attitudes and understandings and reshapes conventional ideas. This might include, for example, the change in attitudes towards care for family members among Turkish migrants living in Germany^6^ (see [7]).

We can certainly observe differences between the first-generation of migrants and the third-generation. For example, we can expect to see differences between a grandfather, who was born in an eastern village in Turkey and came to Germany as a worker (first-generation) and his grandchildren, who were born and raised in Germany (third-generation). His grandchildren are expected to show differences in their biographical background (biographical layer of culture), such as the educational background they received in Germany. However, we can also expect them to eat the same food as a family, to have a basic understanding of national holidays and religious festivals, and what they entail (e.g., visiting the elderly in the family, eating together, etc.) as well as manners and customs (e.g., not talking about money, not speaking loudly in public, eating manners, importance of the family, respecting the elderly, sharing food, etc.), because they share the Turkish cultural background (environmental layer of culture).

### Challenges of Stereotyping and Culturalization: Imposing Further Discrimination?

As suggested above, an appropriate understanding of “culture” as a phenomenon that includes both collective and individual characteristics helps to reconcile the demands of culturally sensitive care and person-centered care. However, there are still challenges arising from stereotyping and culturalization in practice. Stereotype, as defined by Merriam-Webster online Dictionary, is “a standardized mental picture that is held in common by members of a group and that represents an oversimplified opinion, prejudiced attitude, or uncritical judgment” [55]. Stereotypes refer to generalized beliefs about a person as a member of a group, regardless of their own characteristics, or even about a group as it is. As the definition suggests, a stereotype does not necessarily relate to culture. It can be related to gender, age, etc., however, some stereotypes can be ethnic and cultural. Also, stereotypes about gender roles (as in expectations) can be cultural. Culturalization, on the other hand, refers to culture, as in being subject to the influence of culture. As defined by Merriam-Webster online Dictionary, “culturalized” means “deriving from or imposed or conditioned by culture” [56], so that an action or a situation can be explained in terms of one’s cultural affinity. In our context, culturalization could refer to intercultural conflicts in medical settings, where the authority or service provider (i.e., healthcare professionals) and the service users (i.e., the patient and/or their families) have different cultural affinities or backgrounds, and by which a process of othering is induced (see [57]– [58]). Here culturalization has quite a negative connotation. An unwanted request, an unauthorized action or an inappropriate behavior by service users with a migration background may be culturalized by healthcare professionals if these are *attributed to* the service users’ culture or country of origin [58]. These may lead to perceptions of discriminatory behaviors, mistrust of healthcare services, inadequate care for people with migration backgrounds and, therefore, to issues of fairness in the care offered.

The pitfalls of stereotyping and culturalization can result from an essentialist view of culture; that is, if we understand culture as strictly linked and limited to one’s origin, language and religion [59]. On the contrary, if we adopt a flexible approach to culture, where the focus is rather on the individual and the heterogeneity of the individuals, then the concept of culture becomes very vague and empty [59]. It is not a problem to understand culture in a flexible way, nor should it be understood in a negative sense. This is not to say that we do not need to make reference to culture when we think about the provision of care. As shown in the approach above, it would be helpful to have an understanding of culture that lies somewhere between the approaches. It is difficult to make a distinction between person-centered approach and the culturally sensitive approach. Rather, it should be understood that personal values and needs are shaped by one’s cultural background, be it a shared migration experience, growing up in a foreign country within a community from the country of origin, and so on. The person-centered approach and the culturally sensitive approach are then indeed complementary to each other^7^.

If there are culture-specific aspects of dementia care, as argued in the section above, and if culture plays an important role in shaping one’s needs of care, as just argued in the paragraph above, then it would not be wrong to claim that (1) we need categorization in order to clarify and identify the specific needs belonging to each group [60], and (2) we need to institutionalize culturally sensitive (or culturally responsive or tailored) care. This might carry a risk of stereotyping and overgeneralization by grouping people, as some argued (see above). However, such risks do not necessarily arise from adopting a flexible approach to culture. Nevertheless, it is crucial to ask what kind of safeguards are needed in practice in order to avoid stereotyping and culturalization. If the person identifies herself as belonging to a particular group or community, the person would not necessarily feel stereotyped. However, if an outsider were to do such stereotyping, the outsider’s action would be subject to condemnation. It is important to note that individual needs, perceptions and identities of persons should not be ignored when implementing culturally responsive care.

## Conclusion

In this paper, the Turkish community in Germany was taken as an example to demonstrate how both a culturally sensitive and a person-centered approach are needed to develop appropriate care structures for people with migration backgrounds suffering from dementia and for their families. We have also shown how an adequate concept of culture, which includes both environmental and biographical layers, helps to avoid problems of stereotyping and a culturalization of people with migration backgrounds in the context of dementia care. To implement culturally responsive care, awareness-raising (e.g., inclusion of different cultures in healthcare services, recognition of culture) as well as education and training (e.g., knowledge, training, awareness of dementia) of both healthcare personnel (e.g., verbal and non-verbal communication skills, cultural differences in dealing with dementia and misconceptions about the syndrome) and of people of migrant origin affected with dementia (e.g., information about dementia, services and care) is needed [41]. This can be done by using simple language, the first language of the people with migration backgrounds [41]. Understanding where the intercultural conflicts actually lie is an important factor to avoid culturalization of an action, demand or behavior [58]. There is also a need for low-threshold support and counseling in the first language of the people with migration backgrounds [61]. In addition to these points, more people from the same cultural background should work in care facilities. Such staff with cultural affinity would be a valuable resource for training the respective healthcare personnel and identifying specific needs. The institutionalization of culturally sensitive care with continuous supervision would address the inequality of difficult access to the healthcare system for people with migration backgrounds, provide appropriate care for them and encourage them to ask for and receive help.

## Data Availability

No datasets were generated or analysed during the current study.

## Footnotes

1 Looking at the composition of the foreign population by nationality, the largest group reported in 2021 was Turkish nationals, with around 1.46 million people [2]. It is interesting to note that “62.6 per cent of persons with a migrant background have personal experience of migration, i.e., they themselves have migrated to Germany. 37.4 per cent have no personal experience of migration. This shows that a large proportion of persons from the former recruitment countries– or their descendants– were born in Germany: 53.3 per cent of persons with a Turkish, 46.4 per cent of persons with a Moroccan and 43.6 per cent of persons with an Italian migrant background did not migrate to Germany themselves” ([2], p. 150, authors’ translation).

2 Dementia is a term used for several diseases affecting cognitive functions including memory, thinking, orientation, planning, language comprehension and judgment [3–4]. Alzheimer’s disease (AD) is the most common cause of dementia [4]. AD is a gradual, progressive and irreversible neurodegenerative brain disease that causes dependence on others (i.e., formal and informal caregivers) for undertaking activities of daily living. Family members and caregivers of people with dementia play a crucial role in identifying the needs of the people with dementia and representing their interests in terms of care, information, life planning and communication.

3 In the current debate, different terms are used, such as patient-centered care, individual-centered care or even relationship-centered care. In this paper, we will use person-centered care as a key term that encompasses these terms.

4 This means that even though it is not possible to fully comprehend and know what one’s culture requires or demands, healthcare personnel have to put effort into being open and understanding that people may have differing culture-specific needs. However, calling this approach as cultural competence can be misleading as it sounds like simply having competence in communication skills, openness and empathy, as it does not tell us anything specific about the impact of culture on attitudes towards or understanding of illness.

5 Sun-dried food made of curd, tomato and flour, which can be used to make soup. It is one of the traditional dishes that make up the Turkish food culture.

6 As mentioned in the introduction, in Germany, the potential for family care is likely to be reduced in the future as the number of working women with a Turkish migrant background continues to increase. This also signs a change in the role of female family members and expectations regarding care.

7 It is worth emphasizing again that we are not arguing that the culturally sensitive approach and the person-centered approach are very different. However, we do not find *often* an emphasis in the literature on the importance of one’s own cultural influence on the person’s care needs, apart from some of the approaches mentioned above.
